# Bacterial small RNAs may mediate immune response differences seen in respiratory syncytial virus versus rhinovirus bronchiolitis

**DOI:** 10.3389/fimmu.2024.1330991

**Published:** 2024-02-12

**Authors:** Kylie I. Krohmaly, Marcos Perez-Losada, Ignacio Ramos-Tapia, Zhaozhong Zhu, Kohei Hasegawa, Carlos A. Camargo Jr., Brennan Harmon, Janice A. Espinola, Laura Reck Cechinel, Rachael Batabyal, Robert J. Freishtat, Andrea Hahn

**Affiliations:** ^1^ Integrated Biomedical Sciences, The George Washington University, Washington, DC, United States; ^2^ Center for Genetic Medicine Research, Children’s National Research and Innovation Center, Washington, DC, United States; ^3^ Department of Biostatistics and Bioinformatics, Computational Biology Institute, The George Washington University, Washington, DC, United States; ^4^ Centro de Bioinformática y Biología Integrativa, Facultad de Ciencias de la Vida, Universidad Andrés Bello, Santiago, Chile; ^5^ Department of Emergency Medicine, Massachusetts General Hospital, Harvard Medical School, Boston, MA, United States; ^6^ Department of Pediatrics, George Washington University School of Medicine and Health Sciences, Washington, DC, United States; ^7^ Division of Emergency Medicine, Children’s National Hospital, Washington, DC, United States; ^8^ Division of Infectious Diseases, Children’s National Hospital, Washington, DC, United States

**Keywords:** bronchiolitis, respiratory syncytial virus, rhinovirus, *Haemophilus influenzae*, Streptococcus pneumoniae, RNA, extra cellular vesicles

## Abstract

Bronchiolitis, a viral lower respiratory infection, is the leading cause of infant hospitalization, which is associated with an increased risk for developing asthma later in life. Bronchiolitis can be caused by several respiratory viruses, such as respiratory syncytial virus (RSV), rhinovirus (RV), and others. It can also be caused by a solo infection (e.g., RSV- or RV-only bronchiolitis) or co-infection with two or more viruses. Studies have shown viral etiology-related differences between RSV- and RV-only bronchiolitis in the immune response, human microRNA (miRNA) profiles, and dominance of certain airway microbiome constituents. Here, we identified bacterial small RNAs (sRNAs), the prokaryotic equivalent to eukaryotic miRNAs, that differ between infants of the 35^th^ Multicenter Airway Research Collaboration (MARC-35) cohort with RSV- versus RV-only bronchiolitis. We first derived reference sRNA datasets from cultures of four bacteria known to be associated with bronchiolitis (i.e., *Haemophilus influenzae*, *Moraxella catarrhalis*, *Moraxella nonliquefaciens*, and *Streptococcus pneumoniae*). Using these reference sRNA datasets, we found several sRNAs associated with RSV- and RV-only bronchiolitis in our human nasal RNA-Seq MARC-35 data. We also determined potential human transcript targets of the bacterial sRNAs and compared expression of the sRNAs between RSV- and RV-only cases. sRNAs are known to downregulate their mRNA target, we found that, compared to those associated with RV-only bronchiolitis, sRNAs associated with RSV-only bronchiolitis may relatively activate the IL-6 and IL-8 pathways and relatively inhibit the IL-17A pathway. These data support that bacteria may be contributing to inflammation differences seen in RSV- and RV-only bronchiolitis, and for the first time indicate that the potential mechanism in doing so may be through bacterial sRNAs.

## Introduction

Bronchiolitis is a viral lower respiratory infection that manifests as typical cold symptoms (sneezing, coughing, wheezing, fever) (1). Globally, lower respiratory infections are the leading cause of death among young children (<5 years old), accounting for approximately 900,000 deaths annually (2). Bronchiolitis is the leading cause of hospitalization among U.S. infants (2), resulting in about 130,000 hospitalizations every year (3). The effects of bronchiolitis can linger well after recovery; approximately 30% of infants who are hospitalized for bronchiolitis subsequently develop childhood asthma years after hospitalization (4, 5). The 35^th^ Multicenter Airway Research Collaboration (MARC-35) has enrolled over 1,000 infants hospitalized for severe bronchiolitis order to identify risk factors during hospitalization and individuals at a higher risk for developing asthma at 5-years-old and 6-years-old.

Bronchiolitis can be caused by a variety of viruses but respiratory syncytial virus (RSV) and rhinovirus (RV) are two of the most common etiologies (6–8). Although bronchiolitis is considered one disease, there are differences in human metabolites (9), microRNA (miRNA) (10), DNA methylation (11), and immune responses (12–14) depending on virus—specifically, if RSV is the only detected infecting virus (RSV-only bronchiolitis) or if the only detected virus is RV (RV-only bronchiolitis).

Despite bronchiolitis being a viral disease, certain respiratory bacteria such as *Haemophilus influenzae*, *Moraxella catarrhalis*, *Moraxella nonliquefaciens*, and *Streptococcus pneumoniae* are also involved (8, 9, 15–18). Specifically, bronchiolitis caused by RSV tends to be associated with *S. pneumoniae* dominance among other microbiome constituents, while bronchiolitis caused by RV is frequently associated with *H. influenzae* dominance (9, 10). However, the mechanisms by which these bacteria affect bronchiolitis are unknown. While both human metabolites and human miRNAs differed by etiology, bacterial metabolites were not found to be different (9, 10). Additionally, the bacterial analog of miRNAs, small RNAs (sRNAs), were not addressed in these studies. In the present study, we sought to determine if there were any bacterial sRNAs associated with the viral etiology of bronchiolitis.

Bacteria produce regulatory RNAs called small RNAs (sRNAs) which can negatively regulate target transcripts (19, 20). These functional sRNAs can be shuttled between cells through extracellular vesicles (EVs), similar to the transport of eukaryotic miRNAs. These bacterial sRNAs in EVs have been shown not only to be taken up by mammalian cells, but also to be functionally intact after uptake, impact target cell gene expression, and even be incorporated into mammalian miRNA machinery (21–23). While sRNAs from opportunistic airway and periodontal bacteria have been implicated in altering the host immune system (22, 23), it has not been previously described for these species in bronchiolitis.

Herein, we found both previously identified and novel bacterial sRNAs in nasal swab samples from infants with severe bronchiolitis (i.e., bronchiolitis requiring hospitalization) and determined their associations with virus etiology. We hypothesized that *S. pneumoniae* sRNAs would be associated with RSV-only cases and *H. influenzae* sRNAs would be associated with RV-only cases.

## Materials and methods

### Cohort enrollment and nasal swab sample collection

We examined nasal swab RNA-Seq data collected from 589 infants with severe bronchiolitis who were part of the MARC-35 cohort-a multicenter prospective cohort study-comprised of 1,016 infants. These infants (aged <12 months) were enrolled during three consecutive bronchiolitis seasons from 2011-2014, as described previously (10, 24). At index hospitalization, trained investigators collected nasal swab samples using a standardized protocol (25) within the first day of hospitalization, as described previously (10). Nasal swab samples were stored at -80°C until RNA was isolated.

### RNA isolation from nasal swab samples

Total RNA, including small RNA, was isolated from the nasal swab samples using Trizol LS reagent (ThermoFisher Scientific, Waltham, USA) in combination with the Direct-zol RNA Miniprep Kit (Zymo Research, Irvine, USA). RNA quantity was measured with the Qubit 2.0 fluorometer (ThermoFisher Scientific) and RNA quality was assessed with the Agilent Bioanalyzer 2100 (Agilent, Palo Alto, USA) using the RNA 6000 Nano kit.

### Bacterial culture

The type strains of each of the following bacteria were purchased from American Type Culture Collect (Manassas, USA): *H. influenzae* (ATCC 33391) (NCBI RefSeq Assembly Accession GCF_001457655.1), *M. catarrhalis* (ATCC 25238) (GCF_001679005.1), *M. nonliquefaciens* (ATCC 19975) (GCF_900476075.1), and *S. pneumoniae* (ATCC 33400) (GCF_001457635.1). Each species was rehydrated with 7mL of its corresponding broth media (below), incubated overnight in a 37°C, shaking incubator at 225 rotations per minutes (rpm), and stored at -20°C with 15% glycerol.

ATCC 814 GC agar for *H. influenzae* was made with the following ingredients: Dehydrated Culture Media: Gonococcal (GC) Medium Base (Thermo Fisher Scientific, DF0289-17-3), BBL freeze-dried hemoglobin bovine culture media (VWR International, Radnor, USA; 90000-662), and IsoVitaleX Enrichment (Thermo Fisher Scientific, B11876). The medium base and hemoglobin were autoclaved separately (121°C, liquid cycle). When the solutions were cool, the latter solution was mixed into former, along with rehydrated IsoVitaleX, and poured into in 60x15mm petri dishes (VWR International, 25384). Brain heart infusion (BHI) agar (VWR, 90003-040) was made for *M. catarrhalis* and *S. pneumoniae*, set in the same petri dishes. 5% rabbit blood agars (Thermo Fisher Scientific, R01210) were purchased for *M. nonliquefaciens*.

BHI broth (VWR International, 90000-066) was made for each of the bacteria with the following additions: that for *M. nonliquefaciens* was supplemented with 0.7μL beta-nicotinamide adenine diphosphate (Sigma Aldrich, N8285) to every 10mL of broth; that for *H. influenzae* was supplemented with the same amount of beta- nicotinamide adenine diphosphate in addition to 10mL IsoVitaleX to 1L of broth.

Bacterial stock solutions (100-200μL) were inoculated on their respective agar plates and grown for 24-48h (37°C, ambient air). Colony forming units from the agars were collected and used to inoculate respective broth media (50-75mL), which were grown for another 18-24h (37°C, ambient air, shaking at 225rpm).

### Bacterial EV isolation

Broth media were centrifuged at 4,300g for 35 minutes at 4°C to pellet the bacterial cells but leave the bacterial EVs in suspension. The supernatant was separated from the cell pellet by decanting. The cell pellet was resuspended in 250μL PBS.

The supernatant was concentrated to 1-2mL via centrifuging at 4,300g for 35 minutes to 1 hour in 3kDa Amicon Ultra-15 Centrifugal Filter Units (EMD Millipore, Burlington, USA; UFC900324). We added ExoQuick (System Biosciences LLC, Palo Alto, CA; EXOQ20A-1) in a 1:4 (v/v) ratio, incubated at 4°C for 30 minutes, and centrifuged at 1,500g for 30 minutes to precipitate EVs, and resuspended them in 500μL PBS. We used qEV Original resin columns (Izon Science, Christchurch, New Zealand; ICO-35) and the Automatic Fraction Collector (Izon Science, AFC-V1) to purify the EVs, and combined the first three output fractions (total volume = 1.5mL). We again added ExoQuick in a 1:4 (v/v) ratio to these combined fractions incubated at 4°C for 30 minutes, centrifuged at 1,500g for 30 minutes to pellet EVs, and resuspended them in 250μL PBS.

### RNA extraction from bacterial cell pellets and EV pellets

RNA was extracted using a Direct-zol Miniprep kit (Zymo Research, R2053) following the manufacturer’s instructions. Each of the three RNA isolations was performed in triplicate. For EVs and cells of *H. influenzae*, *M. catarrhalis*, and *M. nonliquefaciens*, each of the RNA triplicates was combined across three experiments, ending with three biological RNA replicates for each species. The same procedure was followed for the cells of *S. pneumoniae*, but for *S. pneumoniae* EVs, the biological replicates across three experiments were combined and then re-aliquoted into three technical replicates as the yields were non-uniform from the biological replicates.

### Small RNA-sequencing

All RNA samples were prepared for small RNA-Seq using the PerkinElmer NEXTFLEX® small RNA-Seq v3 kit with Unique Dual Indexes (PerkinElmer, Waltham, USA) and sequenced on an Illumina NovaSeq6000 sequencer using an S2 50bp PE Flowcell (Illumina, San Diego, USA).

### Bioinformatic pipeline for the creation of reference sRNA datasets

**Figure 1:**
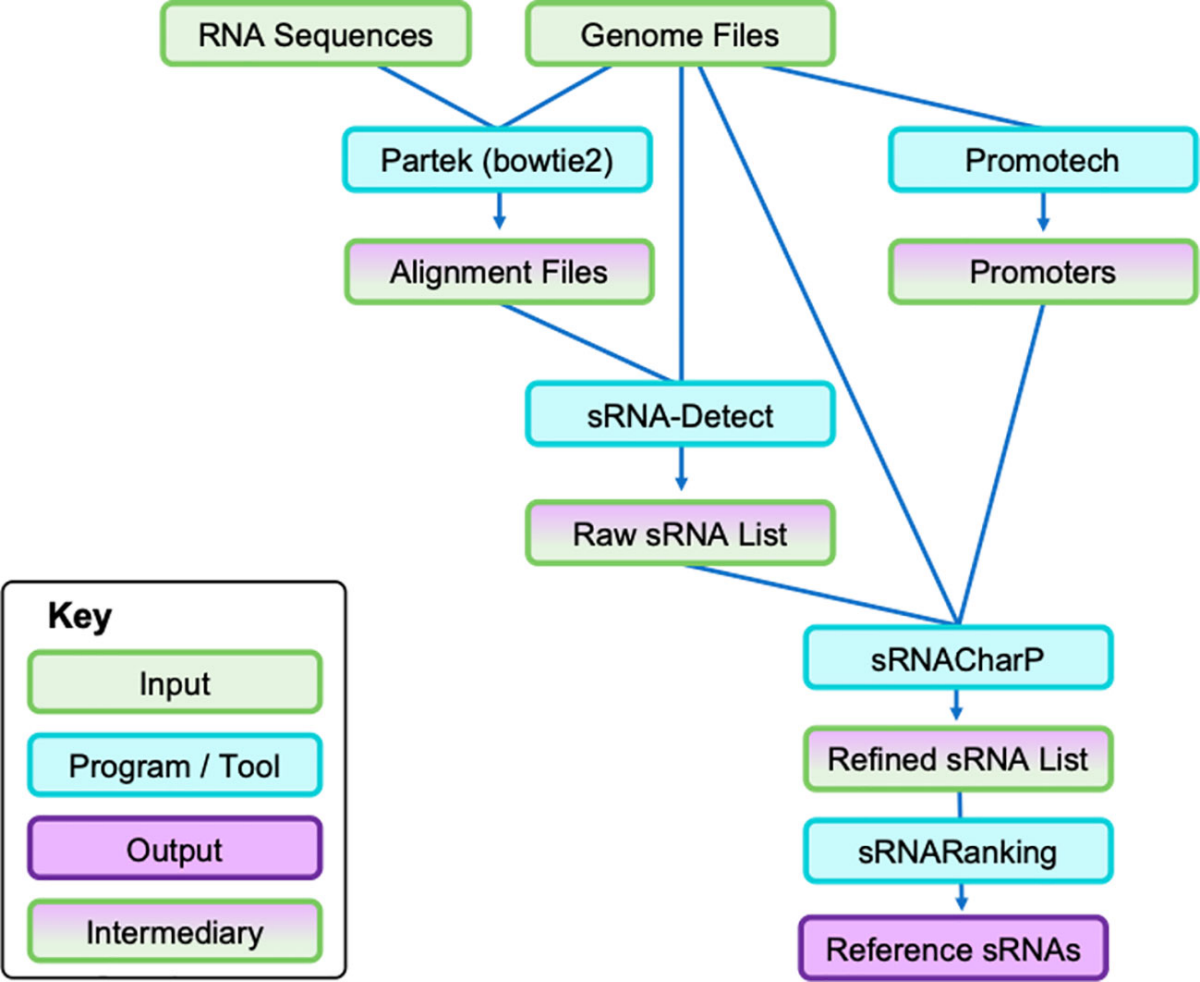
Bioinformatic pipeline to identify bacterial sRNAs from cell pellet RNA and EV pellet RNA. Each species was run independently of each other. Cell and EV pellet RNA were isolated and sequenced. First, genome files (i.e., FASTA sequence and annotation files) were retrieved from NCBI and the RNA sequences were uploaded to Partek to align the sequences to the respective genomes. The subsequent output alignment files and the same genome files were run through sRNA-Detect to generate a broad list of small RNA transcripts. Simultaneously, the genome files were run through Promotech to generate lists of promoters required for sRNACharP. Finally, sRNACharP and sRNARanking were used to create the reference sRNAs for each bacteria from genome files, promoter lists, and output of sRNA-Detect. EV, extracellular vesicle; sRNA, small RNA.

The workflow for the bioinformatic pipeline used is shown in **Figure 1**. First, bacterial small RNA-Seq data were trimmed using Trimmomatic (version 0.33) (26) and uploaded to Partek Flow (Partek Inc., Chesterfield, USA) (27). Genome and annotation files for each bacterial species were retrieved from NCBI (using the NCBI RefSeq Assembly Accession numbers above) and made into Bowtie2 (version 2.5.5) (28) indices in Partek Flow. RNA sequences from the bacterial cells and EVs were aligned to genomes according to species. This output, along with other relevant input files, was run in sRNA-Detect (29), followed by Promotech (30), sRNACharP (31), and sRNARanking (31, 32) to identify novel and known sRNAs for the reference sRNA datasets (see **Supplementary Methods** for more details).

For characterization, sRNA sequences were batch-searched in NCBI (33) and Rfam (34) databases to determine if any sRNAs had previously been identified and to obtain relevant annotations of the sequences. All rho-independent transcription terminators (RITT) sequences in each genome were identified by uploading the entire genome to ARNold (http://rssf.i2bc.paris-saclay.fr/toolbox/arnold/index.php), a web-based service that uses ERPIN (35), RNAmotif (36), and RNAfold (37). BLAST was used to find any overlap between sRNA reference sequences and the RITT sequences identified by ARNold. The reference sRNA datasets and their descriptions can be found in **Supplementary Data 1–4**.

### Determining potential human transcript targets of bacterial sRNAs

To determine potential human transcript targets, the energy of interaction was calculated for all bacterial sRNA sequences and all accessible human 3’ UTR sequences with IntaRNA (38) (see **Supplementary Methods** for more details). Every human 3’UTR sequence available in the Ensembl database (39) that was not “N” or less than 8bp long was examined, following IntaRNA requirements (38) (3,507 unique genes; 19,461 individual 3’UTR sequences; **Supplementary Data 5**). Both the sRNA and 3’UTR sequences were reverse complemented to ensure the interaction between the transcript sequences, would be calculated, and not the gene sequences. The strongest unique interactions between the sRNAs and 3’UTRs were decided by the most negative hybridization energy and the top 250 “potential human transcript targets” of the paired sRNA were considered. We selected 250 targets based on rarefaction curves that showed saturation at that number of targets for the parent processes the targets were involved with via MetaScape (40).

Qiagen’s Ingenuity Pathway Analysis (IPA, version 94302991; https://digitalinsights.qiagen.com/IPA) was used to identify changes in pathway signaling that could be caused by bronchiolitis-associated bacterial sRNAs. This is assuming they act as miRNA and target human transcripts via base-paring at their 3’UTR leading to downregulation of its direct target (the gene of the 3’UTR).

Assuming that sRNAs act as miRNA and target human transcripts via base-paring at their 3’UTR leading to downregulation of its direct target (the gene of the 3’UTR). Qiagen’s Ingenuity Pathway Analysis (IPA, version 94302991; https://digitalinsights.qiagen.com/IPA) was used to identify sRNA targets and subsequent pathway signaling alterations that could be caused by bronchiolitis-associated bacterial sRNAs.

Instead of using miRNA sequences and applying a target filter to identify experimentally validated and predicted miRNA-mRNA interactions, as is typical, we identified the potential targets of the sRNAs through IntaRNA. The sRNAs were not directly put into IPA as they are unavailable in the IPA database. Fold change (FC) and false discovery rate (FDR) of the differentially expressed sRNAs (FC>|1.1| and FDR<0.05) were applied to the respective 250 targets, which were then input into IPA. We conducted a core analysis to identify signaling pathways significantly (p-value < 0.05 by Fisher’s exact test; z-score > |2|) affected by the changes of the differentially expressed sRNAs (**Supplementary Table 1**). For statistically significant pathways, red and green indicated the targets of sRNAs, specifically the those of the sRNAs upregulated in RSV-only bronchiolitis and sRNAs upregulated in RV-only bronchiolitis, respectively. Similarly, blue indicated a predicted pathway activation in RSV-bronchiolitis due to the sRNAs (and predicted inhibition in RV-only cases) while orange indicated the opposite.

### Cytokine analysis

We analyzed cytokine levels from previously described MARC-35 data (41). Briefly, cytokine levels in nasal swab samples from index hospitalization were analyzed using multiplex the Meso Scale Discovery (MSD) electrochemiluminescent V-Plex multiplex immunoassay (Meso Scale Diagnostics, Rockville, MD), on the MESO QuickPlex SQ 120 system (Meso Scale Diagnostics). In the present study, we analyzed IL-1, IL-2, IL-6, and IL-8 measurements by viral etiology (RSV-only versus RV-only).

### Statistical analyses

The cohort used comprised of infants of the MARC-35 cohort with nasal swab sequencing data (n=589). For this analysis, only infants with an RSV infection (RSV-only) or an RV infection (RV-only) as the only detected infecting virus were examined. Differences in patient characteristics were calculated using a Student’s T-test. To search for bacterial sRNAs in the MARC-35 nasal swab sample RNA-Seq data, the sequences in the sRNA reference datasets were converted into Bowtie2 indices (one per species) in Partek Flow. Differential expression analysis was performed via the native implementation of DESeq2 (42) in Partek Flow (normalized by Median ratio) using a Wald test with a Benjamin and Hochberg correction. Cytokine data comparisons used a Mann-Whitney U test with correction, which were computed in Rstudio (v4.1.2), and samples with cytokine levels less than lower limit of detection were removed. All other data were visualized in Rstudio (v4.1.2) using the *tidyverse* (v1.3.2) package.

## Results

### Construction of bacterial sRNA reference datasets

**Figure 2:**
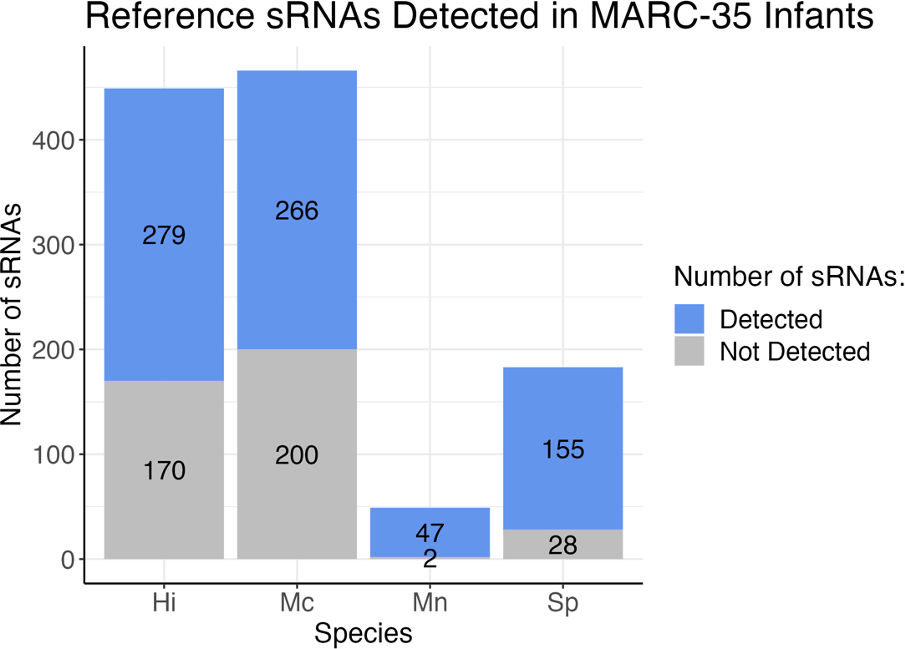
Stacked bar graph depicting the number of reference sRNAs for each species that were detected in the 589 MARC-35 nasal swab samples from infants with severe bronchiolitis. Blue indicates the number of sRNAs in the reference datasets for each bacteria that were detected in human samples, and gray indicates the number of those that were not detected in human samples. Hi, *Haemophilus influenzae*; Mc, *Moraxella catarrhalis*; Mn, *Moraxella nonliquefaciens*; Sp, *Streptococcus pneumoniae*.

We created reference datasets of sRNAs for each species by running small RNA sequencing reads from cultured bacteria and used a multi-program pipeline to bioinformatically identify sRNAs within the bacterial RNA-Seq data (**Figure 1**). Our reference datasets contained 449 putative *H. influenzae* sRNAs, 466 putative *M. catarrhalis* sRNAs, 49 putative *M. nonliquefaciens* sRNAs, and 183 putative *S. pneumoniae* sRNAs (**Figure 2**; see detailed descriptions in **Supplementary Data 1–4**).

We annotated our putative sRNAs by first assessing if they contained rho-independent transcription terminatory (RITT) sequences. Approximately 31-51% of the sRNAs contained RITT sequences depending on the species (**Table 1**). We also searched the sRNA sequences in the BLAST and Rfam databases to obtain annotations. As low as 6% (*M. nonliquefaciens*) and up to 40% (*S. pneumoniae*) of the sRNAs aligned to some known genomic feature. Our reference datasets contained 11 *H. influenzae* sRNAs and 15 *S. pneumoniae* sRNAs that had been previously documented as sRNA sequences in the databases, but no *M. catarrhalis* or *M. nonliquefaciens* sRNAs previously documented as such (**Table 1**). The rest of the sRNAs in the datasets contained sequences that have not been documented as sRNAs and are thus potentially novel.

**Table 1:** Summary of the bacterial reference sRNA datasets.

Species	Total Number of sRNAs in Dataset	Number of sRNAs in Dataset that are:			
		Aligned to RITT Sequences	Found in BLAST and/or Rfam Databases	Previously Known	Novel
Hi	449	215 (48%)	113 (25%)	11 (2%)	438 (98%)
Mc	466	238 (51%)	117 (25%)	0	466 (100%)
Mn	49	15 (31%)	3 (6%)	0	49 (100%)
Sp	183	84 (46%)	73 (40%)	15 (8%)	168 (92%)

RITT, rho-independent transcription terminator; Hi, Haemophilus influenzae; Mc, Moraxella catarrhalis; Mn, Moraxella nonliquefaciens; Sp, Streptococcus pneumoniae.

### Study population

We examined the expression of our reference sRNAs in an analytical cohort of 464 (46%) infants of the 1,016 infant MARC-35 cohort who were hospitalized for bronchiolitis. The analytical cohort was composed of 420 infants with RSV-only and 44 infants with RV-only bronchiolitis. There were no characteristics that differed significantly between the analytic and non-analytic cohorts, except for viral etiology. In the analytical cohort, 41% were female, 47% were non-Hispanic white, 21% were non-Hispanic black, and 29% were Hispanic (**Table 2**). The median age was 3.0 months (IQR = 1.6 - 5.6 months). Additionally, there were no characteristics that differed significantly between the RSV-only group and the RV-only group except that those of the RV-only group were a few months older and weighed more (**Table 2**).

**Table 2:** Patient characteristics.

Characteristic	Analytical Cohort *n =464 (%)*	RSV-Only Cohort *n =420 (%)*	RV-Only Cohort *n = 44 (%)*	Analytical Cohort v RSV-Only Cohort *p-value*	Analytical Cohort v RV-Only Cohort *p-value*	RSV-Only Cohort v RV-Only Cohort *p-value*
Female sex	191 (41)	176 (42)	15 (34)	0.82	0.36	0.32
Race/ethnicity				0.79	0.27	0.23
*Non-Hispanic White*	219 (47)	203 (48)	16 (36)	–	–	–
*Non-Hispanic Black*	96 (21)	86 (21)	10 (23)	–	–	–
*Hispanic*	133 (29)	115 (27)	18 (41)	–	–	–
*Other*	16 (3.4)	16 (3.8)	0 (0)	–	–	–
Parental History of Asthma	144 (31)	131 (31)	13 (30)	0.96	0.83	0.82
Maternal Smoking During Pregnancy	73 (16)	67 (16)	6 (14)	0.92	0.69	0.66
C-section Delivery	170 (37)	152 (36)	18 (41)	0.90	0.61	0.58
Prematurity (32 - 36.9 weeks)	78 (17)	71 (17)	7 (16)	0.97	0.88	0.87
Mostly Breastfeed (3mo)	209 (45)	188 (45)	21 (48)	0.91	0.65	0.62
Previous Breathing Problems				0.40	0.001	< 0.001
*0 Episodes*	385 (83)	357 (85)	28 (64)			
*1 Episode*	64 (14)	52 (12)	12 (27)	–	–	–
*2 Episodes*	15 (3.2)	11 (2.6)	4 (9)	–	–	–
History of Eczema	68 (15)	63 (15)	5 (11)	0.89	0.55	0.52
Ever Attended Daycare	104 (22)	95 (23)	9 (21)	0.9	0.77	0.74
Oxygen Saturation				0.97	0.87	0.86
*<90%*	47 (10)	42 (10)	5 (11)	–	–	–
*90-93%*	69 (15)	64 (15)	5 (11)	–	–	–
*>94%*	339 (73)	305 (73)	34 (77)	–	–	–
Previous Corticosteroid Use	46 (9.9)	39 (9.3)	7 (16)	0.76	0.23	0.17
Previous Antibiotic Use	81 (18)	76 (18)	5 (11)	0.80	0.29	0.25
Corticosteroid Use at Pre-admission Visit	72 (16)	61 (15)	11 (25)	0.68	0.10	0.07
Antibiotic Use at Pre-admission Visit	132 (28)	114 (27)	18 (41)	0.67	0.08	0.05
CPAP	13 (2.8)	12 (2.9)	1 (2.3)	0.96	0.84	0.82
Intubation	16 (3.4)	16 (3.8)	0 (0)	0.77	0.21	0.19
CPAP and Intubation	22 (4.7)	21 (5)	1 (2.3)	0.86	0.45	0.42
Intensive Care	68 (15)	64 (15)	4 (9.1)	0.81	0.31	0.27
Virus				< 0.001	< 0.001	–
*RSV Only*	420 (90.5)	420 (100)	0 (0)	–	–	–
*RV Only*	44 (9.5)	0 (0)	44 (100)	–	–	–
*Any Co-infection*	0 (0)	0 (0)	0 (0)	–	–	–
*Other Virus*	0 (0)	0 (0)	0 (0)	–	–	–
*None*	0 (0)	0 (0)	0 (0)	–	–	–
Characteristic	Analytical Cohort *Median (IQR*)	RSV-Only Cohort *Median (IQR*)	RV-Only Cohort *Median (IQR*)	Analytical Cohort v RSV-Only Cohort *p-value*	Analytical Cohort v RV-Only Cohort *p-value*	RSV-Only Cohort v RV-Only Cohort *p-value*
Age (months)	3.0 (1.6 - 5.6)	2.9 (1.5 - 5.5)	5.0 (2.2 - 6.7)	0.58	0.03	0.01
Body Weight (kg)	6.0 (4.7 - 7.7)	5.9 (4.6 - 7.6)	7.2 (5.5 - 8.6)	0.57	0.03	0.01
Hospital Length of Stay (days)	2 (1 - 3)	2 (1 - 3)	2 (1 - 4)	0.93	0.72	0.70
Respiratory Rate at Presentation (breaths per minute)	48 (40 - 60)	48 (40 - 60)	45 (40 - 58)	0.81	0.32	0.27

RSV, respiratory syncytial virus; RV, rhinovirus. Characteristics are shown as sum and percentage, or median and interquartile range (IQR). P<0.05 are bolded.

### sRNAs associated with viral etiology and their predicted effects in humans

**Figure 3:**
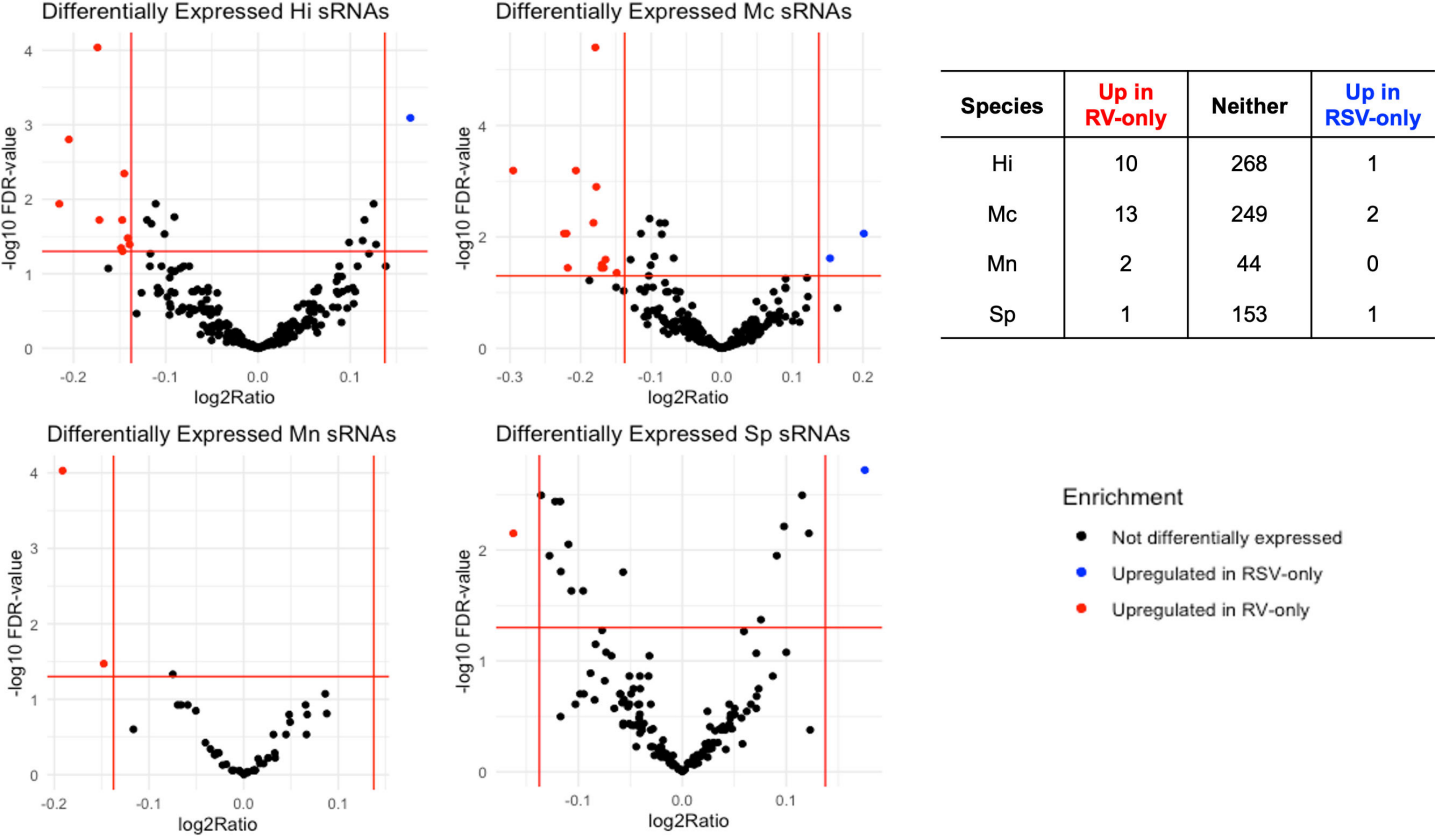
Volcano plots depicting differentially expressed (Fold Change > |1.1|, FDR < 0.05) sRNAs for each bacteria associated with the RV-only group (red dots; n=44) and RSV-only group (blue dots; n=420). Table below shows the number of differentially expressed sRNAs for each group per species. Hi, *Haemophilus influenzae*; Mc, *Moraxella catarrhalis*; Mn, *Moraxella nonliquefaciens*; Sp, *Streptococcus pneumoniae*.

We found 30 sRNAs differentially expressed (FC>|1.1|; FDR<0.05) between RV- and RSV-only bronchiolitis samples, as shown in **Figure 3**, spanning across all four species we looked at. Almost all (26) sRNAs were upregulated in RV-only cases, and thus downregulated in RSV-only cases comparatively (**Figure 3**). Only four sRNAs were upregulated in RSV-only cases, and comparatively downregulated in RV-only cases (**Figure 3**).

All pathways predicted to be significantly different between RV-only cases and RSV-only cases due to the predicted inhibition of transcript targets by the etiology-associated bacterial sRNAs can be found in **Supplementary Table 1**. In this comparative analysis, red and green indicated the targets of sRNAs, which are subsequently downregulated by the respective sRNA, specifically the sRNAs upregulated and associated with RSV-only bronchiolitis and sRNAs upregulated and associated with RV-only bronchiolitis, respectively. Similarly, blue indicated a predicted pathway activation in RSV-bronchiolitis due to the sRNAs (and predicted inhibition in RV-only cases) while orange indicates the opposite.

**Figure 4:**
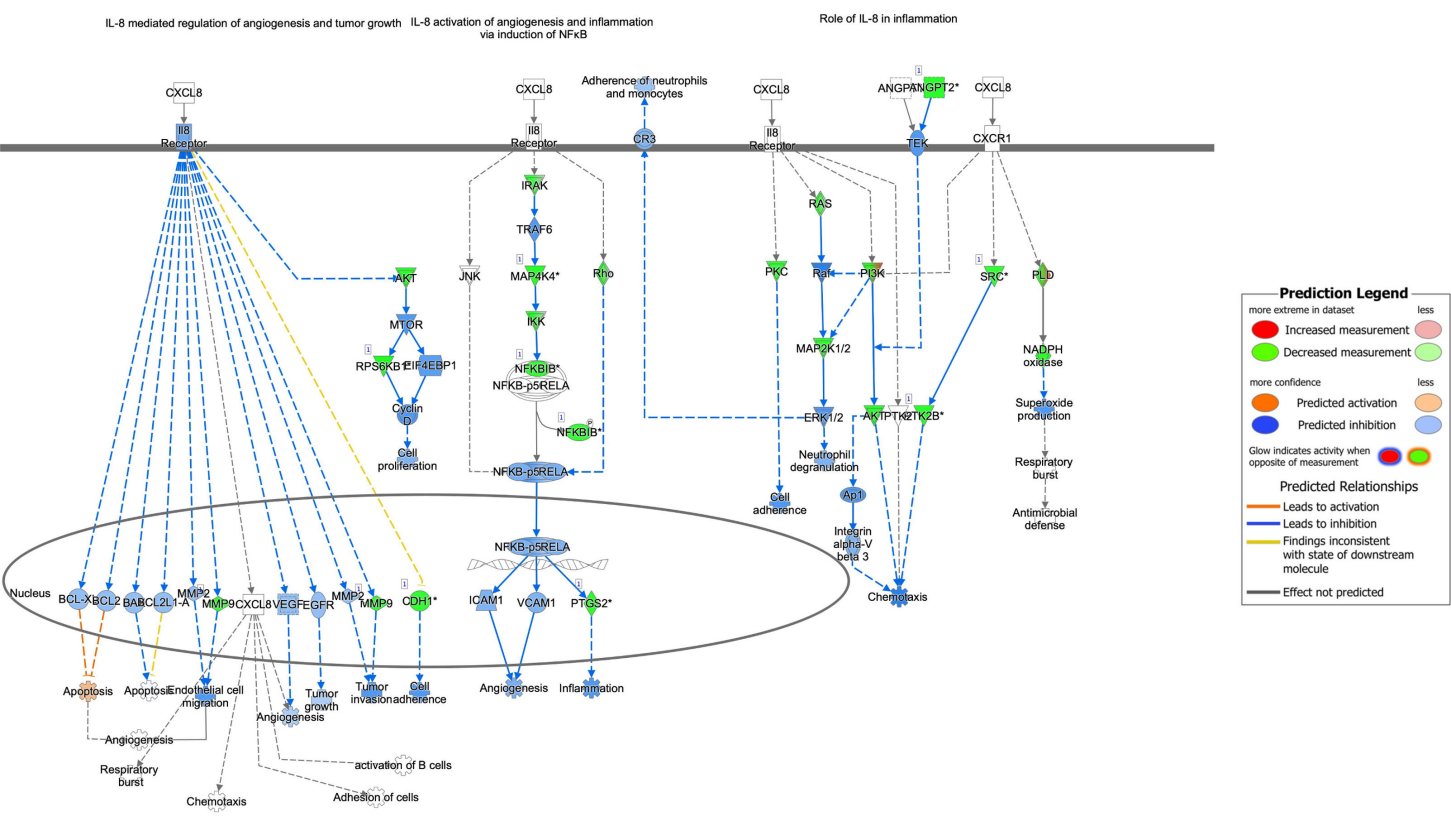
Pathway analysis of sRNA targets affecting the IL-8 pathway in IPA. Compared to RSV-only bronchiolitis, those in green are targets of sRNAs associated with RV-only bronchiolitis, which are assumed to be downregulated in RV-only bronchiolitis, while those in red are targets of sRNAs associated with RSV-only bronchiolitis, which are assumed to be upregulated in RV-only bronchiolitis. Those in blue are predicted to be inhibited, while those in orange are predicted to activated in RV-only bronchiolitis. RSV, respiratory syncytial virus; RV, rhinovirus.

Several components of the interleukin (IL)-8 signaling pathway were targeted by RV-only bronchiolitis-associated bacterial sRNAs (green) (z-score = -5.048; p-value = 0.001) (**Figure 4**). MAP4K4, IKK, and NFKBIB (green), all proteins involved in the pathway signaling of NFkB, were predicted targets of the sRNAs that were upregulated in RV-bronchiolitis, leading to a predicted inhibition of the pathway and subsequent inhibition of inflammation in RV-bronchiolitis compared to RSV-bronchiolitis (**Figure 4**).

**Figure 5:**
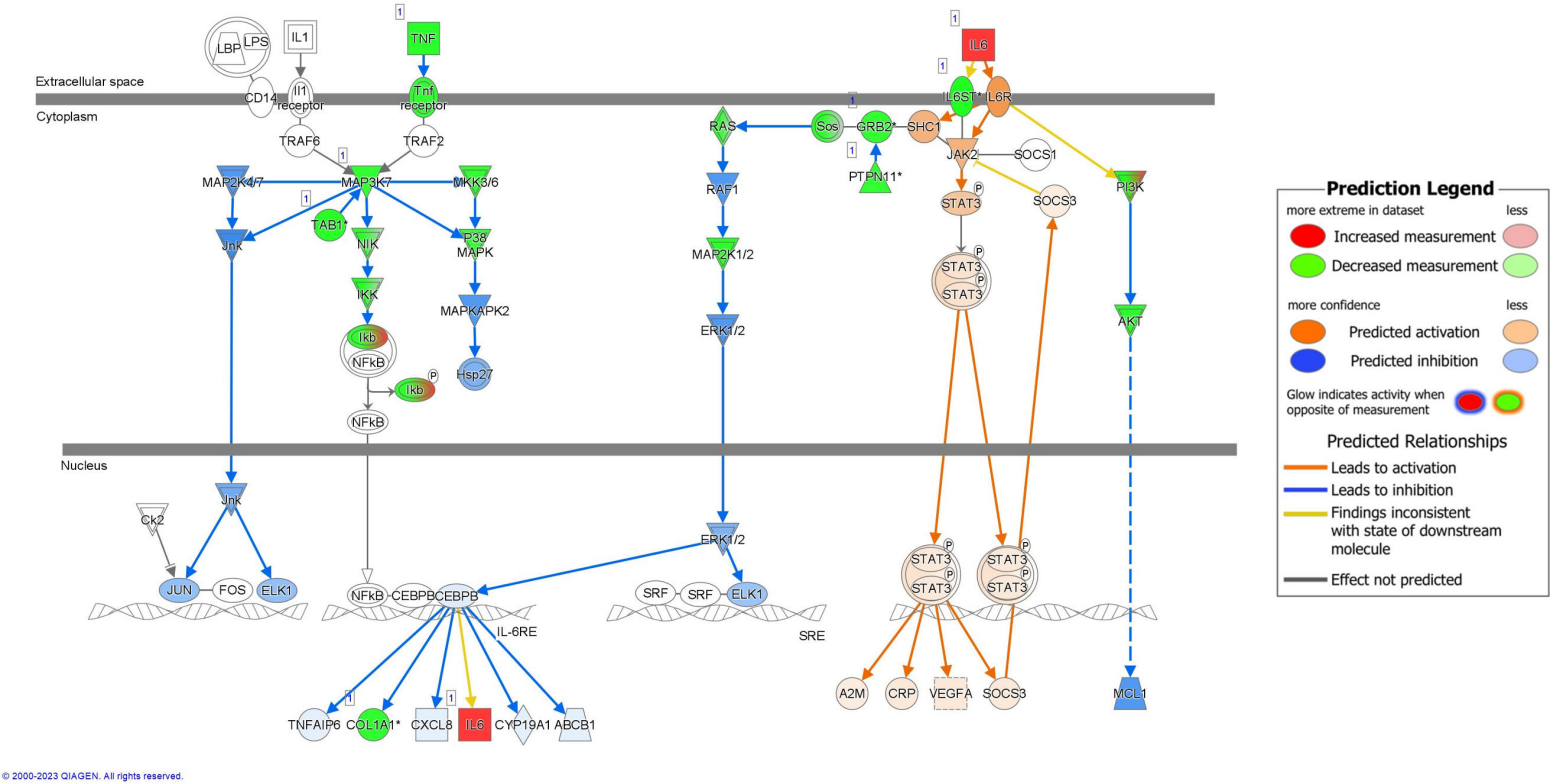
Pathway analysis of sRNA targets affecting the IL-6 pathway in IPA. Compared to RSV-only bronchiolitis, those in green are targets of sRNAs associated with RV-only bronchiolitis, which are assumed to be downregulated in RV-only bronchiolitis, while those in red are targets of sRNAs associated with RSV-only bronchiolitis, which are assumed to be upregulated in RV-only bronchiolitis. Those in blue are predicted to be inhibited, while those in orange are predicted to activated in RV-only bronchiolitis. RSV, respiratory syncytial virus; RV, rhinovirus.

IL-6 signaling was also predicted to be downregulated in RV-only bronchiolitis and upregulated in RSV-only bronchiolitis comparatively (z-score = -4.041; p-value = 0.002) (**Figure 5**). Similarly, proteins involved in NFkB pathway signaling such as TNF, the Tnf receptor, IKK, and others (green) were all predicted targets of sRNAs associated with RV-only bronchiolitis, leading to their predicted downregulation (**Figure 5**). However, Ikb was a predicted target of sRNAs associated with both RSV- and RV-only bronchiolitis (green to red gradient) (**Figure 5**). Despite the predicted downregulation of the pathway, IL-6 was a target of sRNAs associated with RSV-only cases, and therefore there was a higher predicted level of IL-6 and subsequent activation of STAT3 in RV-only bronchiolitis comparatively (**Figure 5**).

We analyzed the levels of IL-6 and IL-8 in a subset of our cohort (RSV-only = 356; RV-only = 33). There were no significant differences in either cytokine by viral etiology (**Supplementary Table 2**).

**Figure 6:**
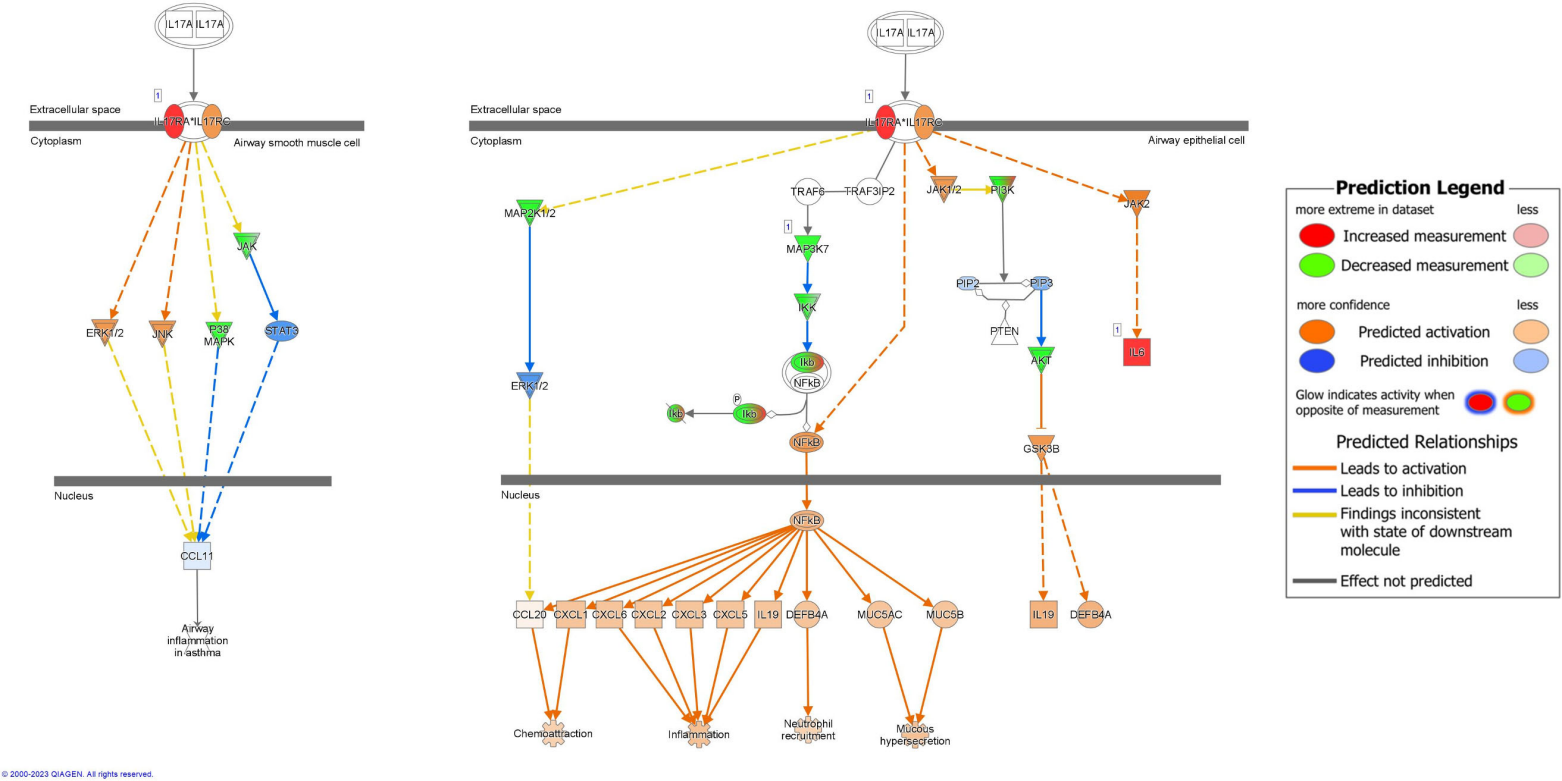
Pathway analysis of sRNA targets affecting IL17A signaling in airway cells. Compared to RSV-only bronchiolitis, those in green are targets of sRNAs associated with RV-only bronchiolitis, which are assumed to be downregulated in RV-only bronchiolitis, while those in red are targets of sRNAs associated with RSV-only bronchiolitis, which are assumed to be upregulated in RV-only bronchiolitis. Those in blue are predicted to be inhibited, while those in orange are predicted to activated in RV-only bronchiolitis. RSV, respiratory syncytial virus; RV, rhinovirus.

Signaling molecules targeted by bacterial sRNAs involved in IL-17A signaling in airway cells were predicted to be overall downregulated in RV-only cases and upregulated in RSV-only cases (z-score = -2; p-value = 0.003) (**Figure 6**). Despite this, the pathway was predicted to result in higher activation of chemoattraction, inflammation, neutrophil recruitment, and mucous hypersecretion in RV-only cases compared to RSV-only cases (**Figure 6**). sRNAs associated with RV-only cases were predicted to target signaling molecules such as MAP3K7, IKK, and Ikb (green), while sRNAs associated with RSV-only cases were predicted to target IL-6 and IL-17RA (**Figure 6**).

Pathways of several cytokines, such as IL-1, IL-2, and IL-23 were predicted to be significantly upregulated in RSV- compared to RV-only bronchiolitis (z-score = -2.84, p-value = 0.003; z-score = -3.357, p-value = 0.004; and z-score = -2.53, p-value = 0.034, respectively) (**Supplementary Table 1**). We also analyzed the levels of IL-1 and IL-2 in a subset of our cohort (RSV-only = 356; RV-only = 33). There were no significant differences in either cytokine by viral etiology (**Supplementary Table 2**).

## Discussion

In this study, we identified numerous novel bacterial sRNAs that were differentially expressed between infants with RSV-only bronchiolitis and infants with RV-only bronchiolitis. These differentially expressed bacterial sRNAs were predicted to interact with several human 3’UTRs in bronchiolitis and potentially lead to the activation of the IL-8 and IL-6 pathways and inhibition of the IL-17A in RSV-only cases, and thus the inhibition of the IL-8 and IL-6 pathways and activation of the IL-17A pathway in RV-bronchiolitis.

IL-8 and IL-6 are two proinflammatory cytokines that have been proposed as potential biomarkers for bronchiolitis severity in several studies (43, 44). While the association with either viral etiology remains unclear, they remain important factors in the pathology of bronchiolitis (43, 45).

Our results indicate that bacterial sRNAs associated with bronchiolitis result in the upregulation of IL-8 and IL-6 pathways in RSV-only cases compared to RV-only cases. The complexity of the decreased predicted measurement of these cytokines, particularly IL-6, with the predicted activation of their signaling pathway seen in RSV-only bronchiolitis is reflected in the literature. One study by Diaz et al. compared cytokine levels of healthy infants to those with RV-only and RSV-only bronchiolitis and showed that those with RSV-only bronchiolitis had significantly higher IL-8 levels than both the healthy individuals and those with RV-only bronchiolitis (45). However, some studies have suggested that IL-8 and IL-6 production are not different between viral groups, consistent with our cytokine measurement data, but between severity (43). Nonetheless, although the difference was not quite significant, infants with RSV-bronchiolitis trended higher in IL-8 and IL-6 production than those with RV-bronchiolitis (43). This, and Diaz’s findings, are consistent with our predictions that IL-8 and IL-6 signaling may be upregulated in RSV-bronchiolitis and comparatively downregulated by bacteria in RV-bronchiolitis.

Morbidity of severe bronchiolitis has repeatedly been associated with a higher risk for developing asthma (4, 5, 46). While IL-17A does not appear to be specifically correlated with either RSV- or RV-bronchiolitis, IL-17A may contribute to airway hyperresponsiveness seen in asthma (47). Here, activation of the IL-17A pathway was associated with RV-only bronchiolitis compared to RSV-only bronchiolitis. Previous studies attempted to create endotypes based on transcriptomic, microbiome, and virus data collected from infants with bronchiolitis (15). Of five endotypes, one was associated with the dominance of *H. influenzae*, RSV/RV co-infection, and upregulation of Th17 pathways (15). Additionally, this endotype was associated with a significantly higher risk of developing asthma (15). While there were significantly different sRNAs of each of the four species between RV-only and RSV-only bronchiolitis, 11 of 30 were of *H. influenzae* origin. It is interesting that bacterial sRNAs are predicted to result in the activation of the IL-17A pathway, specifically in RV-only bronchiolitis, indicating that these sRNAs could be involved in changes to the immune system during and after bronchiolitis that may contribute to asthma.

**Figure 7:**
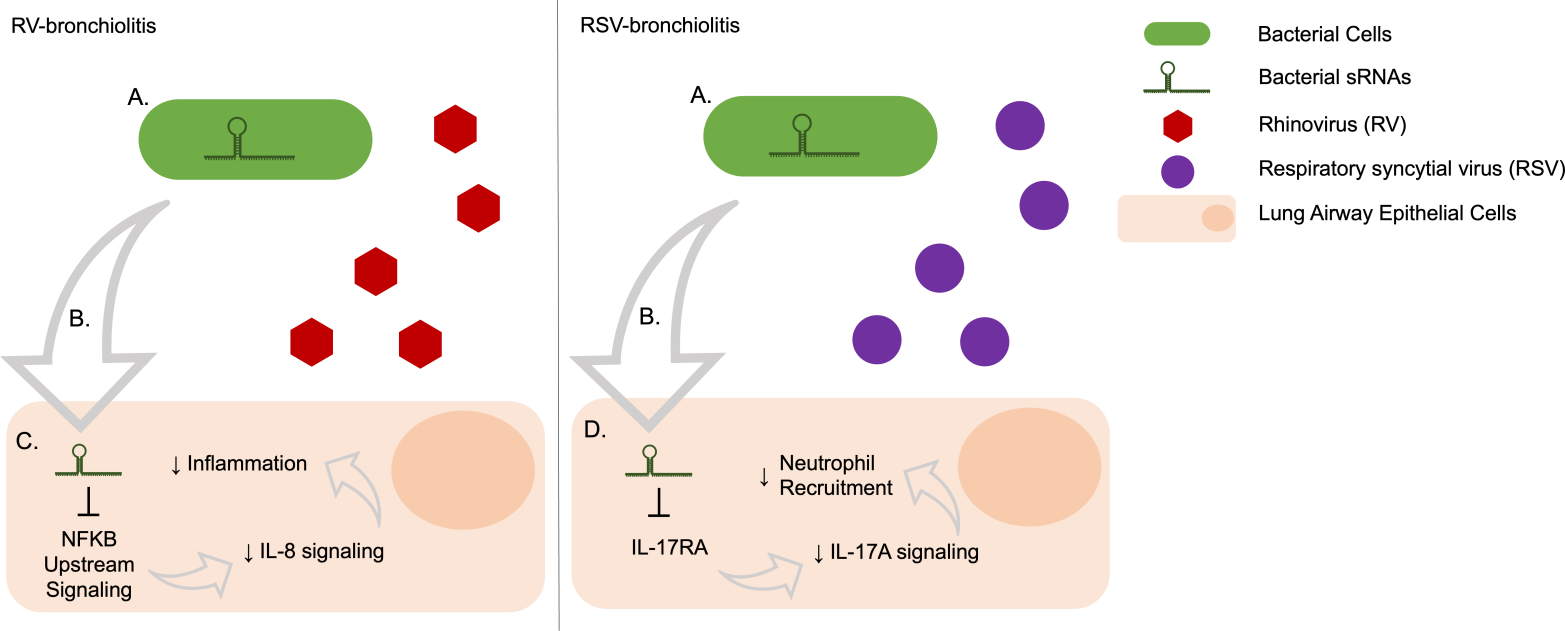
Predicted effects on human gene expression of bacterial sRNAs associated with RV- and RSV-bronchiolitis via target repression. **(A)** Bacteria produce sRNAs, the abundance and presence may be induced or changed depending on the infecting virus. **(B)** Bacterial sRNAs are taken up by human cells, potentially through extracellular vesicles. **(C)** The bacterial sRNAs that are upregulated in RV-bronchiolitis target the IL-8 pathway via NFKB signaling and lead to a decrease in IL-8 signaling, which subsequently leads to a decrease in IL-8 associated inflammation relative to RSV-bronchiolitis. **(D)** The bacterial sRNAs that are upregulated in RSV-bronchiolitis target the IL-17A pathway via IL-17RA and lead to a decrease in IL-17A signaling which subsequently leads to a decrease in neutrophil attraction via IL-17A relative to RV-bronchiolitis.

Additionally, while IL-17A levels in bronchioaveolar lavage fluid (BALF) are not different between mice infected with RV and RSV, mice infected with RV were shown to produce higher BALF levels of neutrophils detected than those with RSV in early infection (48). IL-17A production has been associated with recruiting neutrophils in response to bacterial infection (49, 50). As IL-17A signaling, but not the cytokine itself, and subsequent neutrophil recruitment were predicted to be activated more in RV- than RSV-cases, our data corroborate this and indicate a potential role of RV-bronchiolitis-associated bacterial sRNAs to induce comparatively higher neutrophil activation and recruitment than in RSV-bronchiolitis. On the other hand, signaling of IL-6 and IL-8, common neutrophil attractants, were higher in RSV- than RV-cases. IL-6 was predicted to be a direct target of the bacterial sRNAs in higher abundance in RSV, indicating a direct decrease of the cytokine in RSV cases. Also, although the pathways predicted to be altered by bacterial sRNAs in higher abundance in RSV were related to IL-6 and IL-8 signaling, higher IL-8 signaling was predicted to result in higher general inflammation in RV cases, but it was not predicted to contribute to neutrophil recruitment and activation. Overall, our results are summarized in **Figure 7**.

Several other proinflammatory cytokine pathways were predicted to be affected potential bacterial sRNA modulation, such as IL-1, IL-2, and IL-23, some of which have previously been implicated in bronchiolitis (10, 12, 48, 51, 52) (**Supplementary Table 1**). All these cytokines were predicted to be inhibited in RV-only bronchiolitis compared to RSV-only bronchiolitis due to the bacterial sRNAs (**Supplementary Table 1**). Similar to IL-6 and IL-8, we analyzed the levels of IL-1-beta and IL-2 in nasal swab samples of MARC-35 infants taken at hospitalization, but there were no significant differences in either cytokine by viral etiology (RSV-only versus RV-only). Production of IL-23 in response to Gram-negative bacterial infection can induce production of IL-17A (53, 54), but this is inconsistent with our predictions that IL-17A is a direct target of bacterial sRNAs positively associated with RSV.

Additionally, a notable pathway that has been implicated as different between RSV- and RV-bronchiolitis was NFkB activation by viruses (**Supplementary Table 1**) which was predicted to be upregulated in RSV-only bronchiolitis compared to RV-only bronchiolitis. NFkB is a transcription factor important for immune response and as a mediator of inflammation. RV-bronchiolitis has been associated with a marked increase in NFkB signaling compared to RSV, particularly via human miRNAs. Although the direction of the findings is inconsistent, in both the current study and in earlier work (10), NFkB was predicted to be a target of the regulator RNAs.

There are several potential limitations to this work. First, we use two primary assumptions: 1) that sRNAs can be used like miRNAs and result in the downregulation of its transcript target; and 2) that the transcript targets of our reference sRNAs can be identified by its strongest interactions with human 3’UTR sequences determined by hybridization energy. There is strong evidence to support that sRNAs have been associated with human miRNA machinery (21, 23). Moreover, Furuse et al. found that overexpression of a putative bacterial sRNA in a human cell line was enough to result in downregulation of an artificial target mRNA via a reporter construct (21). Thus, we consider these assumptions to be supported by the literature. Another possible limitation of this work is that no healthy infant controls were used, and thus we cannot make comparisons between viral infection and healthy state. Further, this work focuses on the differences between RSV-only infection versus RV-only infection—the two major viruses of infant bronchiolitis (6–8). There are several viruses that can cause bronchiolitis, and multiple viruses can co-infect an infant to induce bronchiolitis. Thus, the results may not be generalizable to all infants with bronchiolitis. Furthermore, our sample size of infants with RV-only bronchiolitis is much smaller than that of infants with RSV-only bronchiolitis. Another limitation of this study is that our results are computational and require *in vitro* experiments for validation of the interactions and effects of the bacterial sRNAs. Despite our study limitations, we provided novel insights into the mechanisms by which bacteria may be contributing to the clinical differences seen in RSV- and RV-only bronchiolitis.

In summary, we identified many novel sRNAs from several bacterial species that are associated with RV-only and RSV-only bronchiolitis during infancy. In RV bronchiolitis, there is a predicted relative inhibition of IL-8 and IL-6 signaling compared to RSV bronchiolitis, leading to lower predicted inflammation in RV-bronchiolitis (F**igure 7**). Additionally, there is a predicted relative activation of IL-17A in RV bronchiolitis compared to RSV bronchiolitis due to differential expression of bacterial sRNAs, leading to higher predicted neutrophil recruitment in RV-bronchiolitis, and comparatively less so in RSV-bronchiolitis (**Figure 7**). These data indicate that bacteria may be contributing to different mechanisms of inflammation in RSV-only and RV-only bronchiolitis by their sRNAs and may explain in part the heterogeneity seen in infants with bronchiolitis. However, more research is needed to confirm these relationships and identify if these bacterial sRNA-driven alterations also contribute to the heterogeneity seen in asthma risk.

## Data availability statement

The datasets presented in this study can be found in online repositories. The names of the repository/repositories and accession number(s) can be found below: https://www.immport.org/shared/study/SDY2306, SDY2306, https://www.immport.org/shared/study/SDY2157, SDY2157.

## Ethics statement

The studies involving humans were approved by Institutional Review Board of Children’s National Hospital. The studies were conducted in accordance with the local legislation and institutional requirements. The human samples used in this study were acquired from primarily isolated as part of your previous study for which ethical approval was obtained. Written informed consent for participation was not required from the participants or the participants’ legal guardians/next of kin in accordance with the national legislation and institutional requirements.

## Author contributions

KK: Writing – original draft, Writing – review & editing. MP-L: Writing – review & editing. IR-T: Writing – review & editing. ZZ: Writing – review & editing. KH: Writing – review & editing. CC: Writing – review & editing. BH: Writing – review & editing. JE: Writing – review & editing. LRC: Writing – review & editing. RB: Writing – review & editing. RF: Writing – review & editing. AH: Writing – review & editing.
