# Supplementary material for: Bacterial small RNAs may mediate immune response differences seen in respiratory syncytial virus versus rhinovirus bronchiolitis
Source: Front Immunol. 2024 Feb 12;15:1330991. doi: 10.3389/fimmu.2024.1330991 (PMC10895043; doi:10.3389/fimmu.2024.1330991)
Supplement: Supplementary file 6 [file DataSheet_6.docx]

**Supplementary Methods**

**Abbreviations:** Hi, *Haemophilus influenzae*; Mc, *Moraxella catarrhalis*; Mn, *Moraxella nonliquefaciens*; Sp, *Streptococcus pneumoniae*

**Bioinformatic Pipeline**

The genomes .fna/.fasta) and genome annotation files (.gtf) for each of the four bacteria were retrieved from NCBI and uploaded to Partek Flow and Bowtie2 indices created.

Bacterial sRNA sequences were trimmed using Trimmomatic (version 0.33) and uploaded to Partek Flow (Partek Inc., Chesterfield, USA)[1]. Using Bowtie2 (Partek Flow), the RNA sequences from bacterial cells and EVs were aligned according to species, and the subsequent alignment files (.bam) were downloaded. As one of the six paired-end read Hi cell files (forward file for the first Hi cell replicate, Hi1) contained no data (zero bytes), it was excluded from the rest of the analyses. Additionally, the forward and reverse paired-end files for some Hi (HiEV1, HiEV2) and Mc (Mc1, Mc2, Mc3) samples were input and aligned separately due to two downstream errors in sRNA-Detect (potentially due to high input file size).

sRNA-Detect[2] was installed and run via Docker and Nextflow on an Apple M1 MacBook Pro as per the instructions on GitHub (<https://github.com/BioinformaticsLabAtMUN/sRNA-Detect>). One of two main inputs for sRNA-Detect was an alignment file. Genomic feature format (.gff) files (retrieved from NCBI; **Supplementary Table 1**) were the second input for sRNA-Detect. All alignment (.bam) files (from Partek) of one species, regardless of source (cell or EV), with the exception of that of the Hi, were run together in sRNA-Detect. Hi alignment files were separated by source (cell or EV) and had to be run in sRNA-Detect separately from each other at an increased memory in Docker (8GB from 2GB). Each species’ output file (HiA and HiB; Mc; Mn; Sp) from sRNA-Detect (.gtf) of sRNAs was manually converted to BED format (.bed) for use in sRNACharP.

Promotech[3]was installed on a high performance computing Linux system as per instructions on GitHub (<https://github.com/BioinformaticsLabAtMUN/Promotech>), and used to create genome prediction files (.csv) required by sRNACharP. Each individual species’ genome files (.fna/.fasta) was run in Promotech as input, along with the following parameters: default parameters for parsing the whole genome using RF-HOT; threshold set to 0.6 for predicting promoter sequences. While the other three genomes did not need to be altered before being run in Promotech, that for Mn required a couple modifications. As the genome of Mn was retrieved as several contigs, each contig was made into a unique file which was run separately on Promotech. For contigs that had characters other than ACGT (i.e., N), the contigs were split at the illegal character/gap into two files (A, the contig before the characters/gap; and B, that after) and run separately as well. The length of the contig before (length of A) the gap plus the number of characters removed (i.e., the length of N) in the output file was added to the coordinates of the contig split after the gap (added to coordinates of B). Additionally, the regions (lists of contigs as an annotation) in the genome annotation file were removed to yield more than a few sRNAs in subsequent steps. A final singular output file for Mn was created by combining all of the promoter predictions for the Mn contigs in one file.

sRNACharP[4] was installed and run on an Apple M1 MacBook Pro as per instructions on GitHub (<https://github.com/BioinformaticsLabAtMUN/sRNACharP>). Each species’ annotated genomes (.gff) were retrieved from NCBI and converted into BED format (.bed). Each genome (.fna/) was retrieved from NCBI and converted to FASTA format (.fasta). The list of sRNAs (.bed) were run for each species along the three other required files in sRNACharP. Default parameters were used. The output file was a feature table (.tsv), containing the characteristics of each sRNA from the output of sRNA-Detect.

sRNARanking[4,5] was installed and run on an Apple M1 MacBook Pro as per instructions on GitHub (<https://github.com/BioinformaticsLabAtMUN/sRNARanking>). The feature table (.tsv) from sRNACharP was run through sRNARanking via python using default parameters. The output file of sRNARanking contained both the probability of a sequence not being an sRNA and the probability of it being an sRNA (combined, the two probabilities equal 1). Sequences with a probability of being an sRNA above 0.4 were considered probable sRNAs and used to create the reference sRNA dataset for further analysis. Duplicate sRNAs–those with the same exact sRNACharP output sequence–in this dataset were removed.

**Bacterial sRNA Characterization**

IntaRNA[6] was installed on a high performance computing Linux system as per instructions on GitHub (<https://github.com/BackofenLab/IntaRNA>) to determine potential human transcript targets of bacterial sRNAs by calculating the energy of interaction for every combination of bacterial sRNA sequences and human 3’UTR sequences. The two inputs were a list of query sequences (the sRNA sequences) and a list of target sequences (all accessible human 3’ UTR sequences in the Ensembl database). All human 3’ UTR sequences were retrieved from Ensembl BioMart[<http://useast.ensembl.org/biomart/martview/59ef63cacba073d1debc0b6899068a97>] using the Ensembl Genes 110: Human genes (GRCh38.p14) dataset. No filters were used. All sequences run in IntaRNA were reverse-complemented so that the sequence being assessed in IntaRNA was the transcript sequence and not the gene sequence. Additionally, T’s were changed to U’s, and N’s were taken out (these were considered illegal characters in IntaRNA). Each species was run separately. For Hi and Mc, which had a larger dataset of reference sRNAs, the 3’UTR sequences were split into roughly equal six files. All sRNAs were run against all 3’ UTR sequences in IntaRNA.

As one sRNA could interact in several ways with one 3’ UTR, only the lowest energy of interaction (that is, the most energetically favorable or strong interaction) was evaluated for each sRNA-UTR pairing.

**References**

1 Partek Inc. Partek® Flow® (Version 10.0) [Computer software]. https://www.partek.com/partek-flow/. 2020.

2 PEÑA-CASTILLO L, GRÜELL M, MULLIGAN ME, *et al.* DETECTION OF BACTERIAL SMALL TRANSCRIPTS FROM RNA-SEQ DATA: A COMPARATIVE ASSESSMENT. *Biocomputing 2016*. WORLD SCIENTIFIC 2016:456–67. https://doi.org/10.1142/9789814749411_0042

3 Chevez-Guardado R, Peña-Castillo L. Promotech: a general tool for bacterial promoter recognition. *Genome Biol*. 2021;22:318.

4 Eppenhof EJJ, Peña-Castillo L. Prioritizing bona fide bacterial small RNAs with machine learning classifiers. *PeerJ*. 2019;7:e6304.

5 Sorkhian M, Nagari M, Elsisy M, *et al.* Improving Bacterial sRNA Identification By Combining Genomic Context and Sequence-Derived Features. 2022:67–78. https://doi.org/10.1007/978-3-031-20837-9_6

6 Mann M, Wright PR, Backofen R. IntaRNA 2.0: enhanced and customizable prediction of RNA–RNA interactions. *Nucleic Acids Res*. 2017;45:W435–9.
