# Supplementary material for: Bacterial small RNAs may mediate immune response differences seen in respiratory syncytial virus versus rhinovirus bronchiolitis
Source: Front Immunol. 2024 Feb 12;15:1330991. doi: 10.3389/fimmu.2024.1330991 (PMC10895043; doi:10.3389/fimmu.2024.1330991)
Supplement: Supplementary file 7 [file Table_1.docx]

**Supplementary Table 1**. Overview of all processes indicated by IPA that are predicted to be different between RV- and RSV-only cases due to predicted downregulation of target transcripts by the etiology-associated bacterial sRNAs with z-score and p-values.

| Ingenuity Canonical Pathways | z-score | p-value |
| --- | --- | --- |
| Role of Tissue Factor in Cancer | -4.621 | 8.5114E-06 |
| Reelin Signaling in Neurons | -4.849 | 1.2589E-05 |
| AMPK Signaling | -3.286 | 5.2481E-05 |
| VEGF Signaling | -3.13 | 7.7625E-05 |
| Role of NFAT in Cardiac Hypertrophy | -4.439 | 8.9125E-05 |
| Pulmonary Fibrosis Idiopathic Signaling Pathway | -5.077 | 0.00011749 |
| CNTF Signaling | -3.638 | 0.00012589 |
| mTOR Signaling | -2.921 | 0.00014454 |
| HGF Signaling | -3.962 | 0.00016596 |
| NGF Signaling | -4.707 | 0.00016596 |
| Sertoli Cell-Sertoli Cell Junction Signaling | -5.397 | 0.00019055 |
| GM-CSF Signaling | -3.638 | 0.00026303 |
| IGF-1 Signaling | -3.13 | 0.000302 |
| WNK Renal Signaling Pathway | -2.668 | 0.00034674 |
| CD40 Signaling | -2.183 | 0.00035481 |
| Cardiac Hypertrophy Signaling | -5.814 | 0.00038019 |
| Regulation of the Epithelial Mesenchymal Transition by Growth Factors Pathway | -4.11 | 0.00060256 |
| PPAR Signaling | 2.449 | 0.00064565 |
| Natural Killer Cell Signaling | -4.439 | 0.00066069 |
| Opioid Signaling Pathway | -4.128 | 0.00074131 |
| Autophagy | -2.53 | 0.00074131 |
| Death Receptor Signaling | -2.4 | 0.00087096 |
| Role of Osteoclasts in Rheumatoid Arthritis Signaling Pathway | -4.714 | 0.00087096 |
| Toll-like Receptor Signaling | -2.324 | 0.00095499 |
| PI3K/AKT Signaling | -2.785 | 0.00109648 |
| Regulation of eIF4 and p70S6K Signaling | -2.324 | 0.00120226 |
| IL-8 Signaling | -5.048 | 0.00123027 |
| iNOS Signaling | -2.887 | 0.00128825 |
| Thrombin Signaling | -4.7 | 0.00128825 |
| Actin Nucleation by ARP-WASP Complex | -3.873 | 0.00134896 |
| IL-6 Signaling | -4.041 | 0.00147911 |
| ERK/MAPK Signaling | -2.197 | 0.00151356 |
| Melanocyte Development and Pigmentation Signaling | -3.71 | 0.00154882 |
| Neuroinflammation Signaling Pathway | -4.423 | 0.0017378 |
| FLT3 Signaling in Hematopoietic Progenitor Cells | -3.9 | 0.0018197 |
| Serotonin Receptor Signaling | -6.203 | 0.0020893 |
| Integrin Signaling | -5.425 | 0.0020893 |
| RANK Signaling in Osteoclasts | -3.3 | 0.00213796 |
| 14-3-3-mediated Signaling | -2.558 | 0.00223872 |
| Necroptosis Signaling Pathway | -3.286 | 0.00245471 |
| Endocannabinoid Developing Neuron Pathway | -3.545 | 0.00251189 |
| IL-1 Signaling | -2.84 | 0.00275423 |
| Acute Myeloid Leukemia Signaling | -2.668 | 0.00281838 |
| Remodeling of Epithelial Adherens Junctions | -2.53 | 0.00288403 |
| Immunogenic Cell Death Signaling Pathway | -2.982 | 0.00288403 |
| Renal Cell Carcinoma Signaling | -3.207 | 0.00288403 |
| Glioma Signaling | -3.9 | 0.00295121 |
| RHOA Signaling | -3.266 | 0.00295121 |
| ILK Signaling | -3.024 | 0.00316228 |
| Glutaminergic Receptor Signaling Pathway (Enhanced) | -5.357 | 0.00354813 |
| IL-17A Signaling in Fibroblasts | -2.524 | 0.00380189 |
| Actin Cytoskeleton Signaling | -2.874 | 0.00389045 |
| IL-2 Signaling | -3.357 | 0.00389045 |
| Estrogen Receptor Signaling | -4.009 | 0.00446684 |
| MSP-RON Signaling in Cancer Cells Pathway | -3.8 | 0.00446684 |
| Colorectal Cancer Metastasis Signaling | -3.452 | 0.00467735 |
| Ephrin Receptor Signaling | -5.568 | 0.0047863 |
| JAK/STAT Signaling | -2.828 | 0.00512861 |
| ERB2-ERBB3 Signaling | -3.207 | 0.00537032 |
| Hepatic Fibrosis Signaling Pathway | -5.252 | 0.0057544 |
| Neuregulin Signaling | -3.153 | 0.0057544 |
| HIF1α Signaling | -3.55 | 0.00616595 |
| Acute Phase Response Signaling | -4.131 | 0.00660693 |
| Myelination Signaling Pathway | -5.27 | 0.00676083 |
| Gαq Signaling | -3.8 | 0.00707946 |
| Cholecystokinin/Gastrin-mediated Signaling | -4.69 | 0.00724436 |
| IL-7 Signaling Pathway | -2.673 | 0.00812831 |
| HER-2 Signaling in Breast Cancer | -3.43 | 0.00891251 |
| Activin Inhibin Signaling Pathway | -3.667 | 0.00912011 |
| Production of Nitric Oxide and Reactive Oxygen Species in Macrophages | -4.131 | 0.00912011 |
| PDGF Signaling | -3.771 | 0.00954993 |
| p38 MAPK Signaling | -2.357 | 0.00977237 |
| Glioblastoma Multiforme Signaling | -4.158 | 0.00977237 |
| Leukocyte Extravasation Signaling | -3.413 | 0.01 |
| RHOGDI Signaling | 3.674 | 0.01047129 |
| Leptin Signaling in Obesity | -2.333 | 0.01047129 |
| ABRA Signaling Pathway | -2.357 | 0.01071519 |
| Chronic Myeloid Leukemia Signaling | -4.221 | 0.01148154 |
| Role of MAPK Signaling in Inhibiting the Pathogenesis of Influenza | -2.324 | 0.01202264 |
| 4-1BB Signaling in T Lymphocytes | -2.646 | 0.0128825 |
| Synaptogenesis Signaling Pathway | -5.485 | 0.01318257 |
| ID1 Signaling Pathway | -2.744 | 0.01318257 |
| Mouse Embryonic Stem Cell Pluripotency | -3.578 | 0.01318257 |
| Adrenomedullin signaling pathway | -4.7 | 0.01348963 |
| RAC Signaling | -3.273 | 0.01348963 |
| Chemokine Signaling | -4 | 0.01348963 |
| PTEN Signaling | 3.128 | 0.01348963 |
| Sphingosine-1-phosphate Signaling | -2.4 | 0.01412538 |
| Growth Hormone Signaling | -2.496 | 0.01412538 |
| NF-κB Activation by Viruses | -3 | 0.01513561 |
| Endothelin-1 Signaling | -4.017 | 0.01548817 |
| LPS-stimulated MAPK Signaling | -3.153 | 0.0162181 |
| Aldosterone Signaling in Epithelial Cells | -2.84 | 0.01737801 |
| Ribonucleotide Reductase Signaling Pathway | -2.646 | 0.01862087 |
| Non-Small Cell Lung Cancer Signaling | -2.309 | 0.01862087 |
| ERBB Signaling | -3.771 | 0.01862087 |
| Cardiac Hypertrophy Signaling (Enhanced) | -4.95 | 0.01862087 |
| Neuropathic Pain Signaling in Dorsal Horn Neurons | -3.441 | 0.01905461 |
| Senescence Pathway | -4.422 | 0.01949845 |
| Signaling by Rho Family GTPases | -4.7 | 0.02041738 |
| Oxytocin Signaling Pathway | -5.032 | 0.02238721 |
| Prolactin Signaling | -3 | 0.02290868 |
| Macropinocytosis Signaling | -2.309 | 0.02570396 |
| Orexin Signaling Pathway | -4.11 | 0.02570396 |
| Thrombopoietin Signaling | -3.051 | 0.02570396 |
| Role of MAPK Signaling in Promoting the Pathogenesis of Influenza | -2.982 | 0.02570396 |
| Fc Epsilon RI Signaling | -3.13 | 0.02818383 |
| Glioma Invasiveness Signaling | -2.496 | 0.03090295 |
| IL-33 Signaling Pathway | -2.785 | 0.03235937 |
| Pyridoxal 5'-phosphate Salvage Pathway | -3.464 | 0.03235937 |
| Paxillin Signaling | -3.5 | 0.03388442 |
| RAR Activation | -4.648 | 0.03388442 |
| IL-23 Signaling Pathway | -2.53 | 0.03388442 |
| EIF2 Signaling | -2.4 | 0.03548134 |
| STAT3 Pathway | -2.982 | 0.03548134 |
| FGF Signaling | -3 | 0.03548134 |
| ERBB4 Signaling | -3.051 | 0.03630781 |
| Macrophage Alternative Activation Signaling Pathway | -3.9 | 0.03981072 |
| Lymphotoxin β Receptor Signaling | -2.714 | 0.04168694 |
| ERK5 Signaling | -3.207 | 0.04168694 |
| CCR3 Signaling in Eosinophils | -2.111 | 0.04168694 |
| GP6 Signaling Pathway | -4.025 | 0.04265795 |
| Phosphatidylglycerol Biosynthesis II (Non-plastidic) | -2.646 | 0.04265795 |
| Antioxidant Action of Vitamin C | 2.183 | 0.04265795 |
| PAK Signaling | -3.5 | 0.04677351 |
| Apelin Endothelial Signaling Pathway | -2.982 | 0.04786301 |
| Role of PKR in Interferon Induction and Antiviral Response | -2.132 | 0.04897788 |
| IL-17A Signaling in Airway Cells | -2 | 0.003388 |
| Pancreatic Adenocarcinoma Signaling | -2 | .007413 |
| NAD Biosynthesis II (from tryptophan) | -2 | .010471 |
