# Supplementary material for: Bacterial small RNAs may mediate immune response differences seen in respiratory syncytial virus versus rhinovirus bronchiolitis
Source: Front Immunol. 2024 Feb 12;15:1330991. doi: 10.3389/fimmu.2024.1330991 (PMC10895043; doi:10.3389/fimmu.2024.1330991)
Supplement: Supplementary file 8 [file Table_2.docx]

**Supplementary Table 2**. Levels of IL-1, IL-2, IL-6, and IL-8 in a subset of the MARC-35 nasal swab samples analyzed at index hospitalization.
